# Multiscale Control of Nanofiber-Composite Hydrogel for Complex 3D Cell Culture by Extracellular Matrix Composition and Nanofiber Alignment

**DOI:** 10.34133/bmr.0032

**Published:** 2024-05-29

**Authors:** Cholong Choi, Eunhye Yun, Minju Song, Jiyun Kim, Jae Sung Son, Chaenyung Cha

**Affiliations:** ^1^ Center for Multidimensional Programmable Matter, Department of Materials Science and Engineering, Ulsan National Institute of Science and Technology (UNIST), Ulsan 44919, Republic of Korea.; ^2^ Department of Chemical Engineering, Pohang University of Science and Technology (POSTECH), Pohang, Gyeongsangbuk-do 37673, Republic of Korea.

## Abstract

In order to manipulate the complex behavior of cells in a 3-dimensional (3D) environment, it is important to provide the microenvironment that can accurately portray the complexity of highly anisotropic tissue structures. However, it is technically challenging to generate a complex microenvironment using conventional biomaterials that are mostly isotropic with limited bioactivity. In this study, the gelatin-hyaluronic acid hydrogel incorporated with aqueous-dispersible, short nanofibers capable of in situ alignment is developed to emulate the native heterogeneous extracellular matrix consisting of fibrous and non-fibrous components. The gelatin nanofibers containing magnetic nanoparticles, which could be aligned by external magnetic field, are dispersed and embedded in gelatin-hyaluronic acid hydrogel encapsulated with dermal fibroblasts. The aligned nanofibers via magnetic field could be safely integrated into the hydrogel, and the process could be repeated to generate larger 3D hydrogels with variable nanofiber alignments. The aligned nanofibers in the hydrogel can more effectively guide the anisotropic morphology (e.g., elongation) of dermal fibroblasts than random nanofibers, whereas myofibroblastic differentiation is more prominent in random nanofibers. At a given nanofiber configuration, the hydrogel composition having intermediate hyaluronic acid content induces myofibroblastic differentiation. These results indicate that modulating the degree of nanofiber alignment and the hyaluronic acid content of the hydrogel are crucial factors that critically influence the fibroblast phenotypes. The nanofiber-composite hydrogel capable of directional nanofiber alignment and tunable material composition can effectively induce a wide array of phenotypic plasticity in 3D cell culture.

## Introduction

Native extracellular matrix (ECM) is highly heterogeneous, consisting of both fibrous collagen fibrils crosslinked with non-fibrous proteoglycans [1,2]. In addition to compositional diversity, morphological features of ECM are also heavily dependent on the type of tissue. This dynamic presentation of both chemical and physicomechanical characteristics of ECM is crucial in mediating various cellular phenotypes, such as spreading, differentiation, and migration, and guiding tissue texture and specific organ morphogenesis at various stages of development, disease progression, and wound healing [3–5].

Hydrogels have been widely utilized as platforms to culture cells and tissues for biomedical applications, as their typical physicomechanical properties, such as tunable elasticity and high water content via swelling, can approximate those of native ECM [6–10]. The bioactive properties of hydrogels can also be tailored to present various cell-responsive moieties, such as cell adhesive peptides derived from ECM proteins, and soluble factors necessary for cell adhesion and subsequent phenotypic changes [11–14]. However, most conventional methods to engineer hydrogels involve the crosslinking of monomers or macromers at a molecular level, which leads to an isotropic polymeric network with limited porosity. As a result, most of conventional hydrogels are unable to recapitulate the heterogeneous and anisotropic nature of native ECM, only being able to generate a limited scope of phenotypic plasticity of the cells.

Nanocomposite hydrogels incorporating nanofibers as a filler have received considerable attention in recent years as a biomimetic approach to recreate the cellular microenvironment that can more accurately recreate the complexity of heterogeneous tissue architecture [15–20]. Nanofibers prepared by electrospinning have been widely utilized in tissue engineering, as the meshwork of interlaid nanofibers has been shown to be quite effective as scaffolds, promoting cell adhesion and growth [21]. Moreover, anisotropically configured nanofibers, such as those aligned in a certain direction, could guide the cells to acquire elongated morphology and migrate in the direction of the alignment, which is highly desired for muscle and nerve cells [22–25]. Nanofibers that are cut short enough, usually micrometer scale in length, can be safely dispersed in hydrogels without aggregation or sedimentation, so their presence has been shown to promote the mechanical properties of resulting nanocomposite hydrogels without adversely affecting their permeability, which has long been a predicament for conventional hydrogels displaying an inverse relationship between the mechanics and diffusion [13,26,27]. Furthermore, the spatial distribution of nanofibers within the hydrogel can be controlled to emulate tissue anisotropy using various fabrication methods. For example, shear force is often introduced to the precursor solution to align nanofibers prior to hydrogel fabrication [28,29]. For hydrogels with more balanced viscoelasticity, tensile force can be applied to induce the alignment of nanofibers within the hydrogels [30–32].

Magnetic nanoparticles (MNPs) are actively explored in biomedical engineering especially for their ability to respond to external magnetic field, such as photothermal therapy and contrast agents for magnetic resonance imaging [33–35]. With the ability to move under the magnetic field, MNPs have been increasingly adopted to control the alignment of nanofibers in a refined manner [36–40]. Since MNPs can be easily embedded into a wide array of polymers, the short nanofibers embedded with MNPs have been shown to undergo alignment by the MNP dragging the nanofibers in a single direction under magnetic force. This approach has the critical advantage of more precise nanofiber alignment by controlling the magnitude and direction of the magnetic field. In addition, it can be applied to a wide range of nanofibers, as long as the miscibility between the MNP and the nanofiber is guaranteed.

Taking advantage of this strategy, herein, photocrosslinkable gelatin nanofibers embedded with MNPs were incorporated and magnetically aligned in a hydrogel to control various phenotypes of encapsulated cells. The aqueous-dispersible and magnetically active gelatin nanofibers were generated by electrospinning gelatin and MNPs, followed by ultrasonic cutting into micrometer length, and chemical crosslinking to prevent dissolution in aqueous media [15,16]. The concentration of MNP in nanofiber was controlled to maximize the nanofiber movement and alignment. Unlike previous studies that have mostly focused on the nanofiber alignment itself, this study broadly explores the consequence of manipulating both nanofiber alignment and physicomechanical properties of hydrogels on the encapsulated cells. This was accomplished by incorporating and aligning gelatin nanofibers within a photocrosslinked hybrid hydrogel consisting of methacrylate-functionalized gelatin and hyaluronic acid, emulating the native tissue consisting of collagen fibrils crosslinked with proteoglycans. Since the photocrosslinking and magnetic-induced nanofiber alignment can be repeated, it was also possible to generate a complex heterostructure consisting of hydrogels having different nanofiber alignments. The combined effects of hydrogel composition and the nanofiber alignment on encapsulated dermal fibroblasts were explored to demonstrate the consequence and importance of multiscale control of a 3-dimensional (3D) microenvironment in deriving a wide range of cellular responses.

## Materials and Methods

### Synthesis and surface modification of MNP

Superparamagnetic iron oxide (Fe_3_O_4_) nanoparticles as MNPs were synthesized following a previously established method (the detailed synthetic procedure is provided in the Supplementary Materials) [41]. As-fabricated MNP was then functionalized with amine. Briefly, 1 mg of MNP was dispersed in 10 ml of toluene. (3-Aminopropyl)trimethoxysilane (APTMS, 90 μl; Sigma-Aldrich) was slowly added and stirred for 72 h at room temperature, during which the oleic acid on the MNP surface was exchanged with APTMS.

The amine-functionalized MNP (NH_2_-MNP) was conjugated with gelatin via 1-ethyl-3-(3-dimethylaminopropyl) carbodiimide (EDC) coupling [42]. First, 10 ml of 0.2% (w/v) gelatin (from porcine skin, Sigma-Aldrich) was mixed with 2 ml of 26 mM EDC (Sigma-Aldrich) and 10 mM *N*-hydroxysuccinimide (NHS, Sigma-Aldrich) in MES (2-(N-morpholino)ethanesulfonic acid) buffer (pH 6.4) and stirred overnight at room temperature to activate the gelatin with NHS ester. Then, 20 ml of 0.2% (w/v) NH_2_-MNP solution was slowly added to the mixture and further stirred for 24 h. The resulting gelatin-functionalized MNP (G-MNP) was washed with deionized water several times and collected by centrifugation.

### Preparation of MNP-laden aqueous-dispersible nanofibers

The nanofiber precursor solution was prepared by dispersing G-MNP in methacrylate-functionalized gelatin (MGel) solution in 2,2,2-trifluoroethanol/deionized water (7:3 volume ratio). The concentration of MGel was 16% (w/v), while that of SPION was controlled up to 1% (w/v). The solution was sonicated in a pulse mode (5 s on, 5 s off, output power: 65 W, frequency: 20 kHz, VCX130, Sonic) for 10 min to induce homogeneous dispersion. The solution was loaded into a syringe with a 26 G needle and ejected at the flow rate of 1 ml h^–1^. Twelve kilovolts was applied between the needle and an aluminum rotating drum collector at 1,000 rpm, resulting in aligned nanofiber deposition. The fiber mat was dried in vacuo for 24 h.

The dried fiber mat was soaked in EDC (2 mM)/NHS (2 mM) in 95% ethanol for 48 h with gentle shaking to crosslink the nanofibers and prevent their dissolution in aqueous media [16]. The mat was washed 3 times with ethanol and isopropanol and dried in vacuo for 30 min. In order to develop short nanofibers, after drying, the nanofibers were immersed in isopropanol and were sonicated in the same pulse mode for MNP dispersion. The diameter and length of nanofibers were measured from scanning electron microscopic (S-4800, Hitachi) images. The presence of MNP in the nanofiber was further confirmed with transmission electron microscopy (JEM-1400, JEOL) (Fig. S1).

### Magnetic-induced nanofiber alignment in hydrogel

A gel precursor solution consisting of 0.5% (w/v) MNP-nanofibers dispersed in a prepolymer solution consisting of MGel, methacrylate-functionalized hyaluronic acid (MHA), and 0.2% (w/v) Irgacure 2959 was placed between 300-μm spacers. The MNP contents of nanofibers were 0.75, 1.5, 3, and 6 wt%. The concentrations of MGel and MHA were controlled up to 8% (w/v) and 0.5% (w/v), respectively. Two neodymium magnets (410 mT, JJTOOLS Co., Korea) were placed in between the precursor solution up to 60 s to obtain nanofiber alignment. Then, the solution was irradiated with UV for 60 s (800 mW cm^–1^, Omnicure S1500) to induce hydrogel formation. As a control group, the hydrogels only made with MGel at different concentrations, 8%, 10%, and 12% (w/v), were also used.

To create a larger hydrogel having multiple different nanofiber alignments, the photocrosslinking process was repeated layer by layer with each layer having a different direction of nanofiber alignment. Briefly, the precursor solution was placed in between 2 glass plates with a 0.1-mm spacer, and magnetic field was applied to induce the nanofiber alignment. After photocrosslinking, fresh precursor solution was placed on top of the cured hydrogel immediately afterwards with an additional spacer and magnetic field was applied in a different direction and photocrosslinked. This layer-by-layer process was repeated to generate a multilayered hydrogel with 3D heterogeneous nanofiber alignment.

For evaluating the efficiency of nanofiber alignment by the magnetic field, the angular distribution of nanofibers was obtained and analyzed [37,43]. Briefly, the fluorescent image of nanofibers within hydrogel was first obtained with a scanning confocal microscope (FV1000, Olympus). The angles of nanofibers were measured using an image-processing software (ImageJ, a free software from https://imagej.nih.gov/ij/). The maximum degree of rotation in which the nanofiber was oriented parallel to the external magnetic field was designated as zero degree. The histogram of the angular variation of nanofibers was fitted to the Gaussian distribution, and the full width at half maximum (FWHM) was determined (Origin 2020, OriginLab).

### In vitro characterization

#### Cell viability

Primary human adult dermal fibroblasts (NHDF-Ad, Lonza) were used in this study. The cells were cultured in Dulbecco,s modified Eagle,s medium supplemented with 1% penicillin/streptomycin and 10% fetal bovine serum (Thermo Fisher). The fibroblasts were dispersed in the precursor solution (1 × 10^6^ cells ml^–1^) and incubated for 2 h with gentle shaking to induce cell adhesion to nanofibers prior to hydrogel formation. The precursor solution was then exposed to an external magnetic field, as described above, to induce the nanofiber alignment. After hydrogel was made by photocrosslinking, the cell-laden hydrogels were immersed in the cell culture medium and continuously cultured at 37 °C under 5% CO_2_.

To evaluate the viability of encapsulated cells, the cells were fluorescently labeled with calcein-AM and ethidium homodimer-1 to distinguish live (green) and red (dead) cells, respectively (LIVE/DEAD Cell Viability/Cytotoxicity Kit, Thermo Fisher) [44]. The cells were visualized under a fluorescence microscope (XDS-3FL, Optika).

#### Immunocytochemistry

Actin filaments and nuclei of the cells were visualized by fluorescent labeling [15]. The samples were fixed with 4% formaldehyde for 30 min, permeabilized with 0.1% Triton X-100 for 1 h, and blocked with 2% bovine serum albumin for 2 h in phosphate buffered saline (PBS). The samples were then treated with rhodamine-labeled phalloidin (1:1,000 dilution, Abcam) and 4′,6-diamidino-2-phenylindole (Sigma-Aldrich) to label and visualize the actin and nuclei, respectively. Samples were washed 3 times with PBS after each step and the entire process was performed at room temperature. The fluorescent images were obtained with a scanning confocal microscope (FV1000, Olympus). The same procedure was followed for immunofluorescent labeling of alpha smooth muscle actin (α-SMA), using mouse α-SMA monoclonal antibody (1:1,000 dilution, Invitrogen) as the primary antibody and Alexa fluor 488 goat anti-mouse antibody as the secondary antibody (1:1,000 dilution, Invitrogen). The amount of α-SMA was estimated by quantifying the intensity of fluorescence from the images via counting the pixels in each cell using ImageJ (a free image analysis software, https://imagej.nih.gov).

#### Quantitative real-time PCR

The total RNA was isolated from fibroblasts in hydrogel, using TRIzol Reagent (Thermo Fisher Scientific). RT-PCR was performed with the total RNA (500 ng) to synthesize complementary DNA with Omniscript RT kit (Qiagen) using oligo-dT primers. Quantitative real-time PCR was performed on LightCycler480 instrument II using SYBR Green I Master (Roche). The mRNA primer sequences for α-SMA (*ACTA2*) and GADPH (*GADPH*) are as follows: ACTA2-F (5′-GCTTTGGCTAGGAATGATTTGG-3′), ACTA2-R (5′-GCTTTGGCTAGGAATGATTTGG-3′), GADPH-F (5′-GTGGACCTGACCTGCCGTCT-3′), and GADPH-R (5′-GGAGGAGTGGGTGTCGCTGT-3′). The experiments were performed in triplicate and normalized to the housekeeping gene (GADPH). Expression levels were compared using the comparative Ct method.

### In vivo characterization

#### Hydrogel implantation

Six-week-old male Sprague-Dawley (SD) rats, purchased from Hyochang Science (Daegu, South Korea), were used for in vivo studies. All animal experiments performed for this study were carried out in compliance with Institutional Animal Care and Use Committee of Ulsan National Institute of Science and Technology (#UNISTIACUC-23-05). A 2-cm incision was made in the mediodorsal skin of the rat, and a lateral subcutaneous pocket was prepared. Each condition of hydrogel disk samples (5 mm in diameter, 1 mm in thickness) was implanted under sterile conditions. At designated time intervals (7 and 14 days), the rats (*n* = 3 for each day) were sacrificed. Then, the skin tissue samples around the implant areas were excised and preserved in 4% paraformaldehyde for histopathological analysis.

#### Histology

The hydrogel implanted skin tissue samples were paraffin embedded, sectioned (6 μm thickness), and mounted on glass slides. Then, a standard hematoxylin and eosin staining was performed on the sectioned tissue samples and visualized under an inverted optical microscope (CKX53, Olympus).

### Statistical analysis

Mean and standard deviation values from multiple independent experiments were reported in this study (*n* = 10 for material characterization, *n* = 6 for in vitro studies). Outliers that were beyond upper and lower fences were omitted. Statistical significance of differences between 2 conditions was assessed using Student’s *t*-test. Statistical significance of difference between multiple conditions was evaluated by one-way ANOVA followed by Tukey,s post hoc test (Microsoft Office Excel). *P* values below 0.05 were considered statistically significant and are reported here.

## Results

### Physicomechanical properties of MNP-laden, aqueous-dispersible gelatin nanofiber

**Fig. 1.**
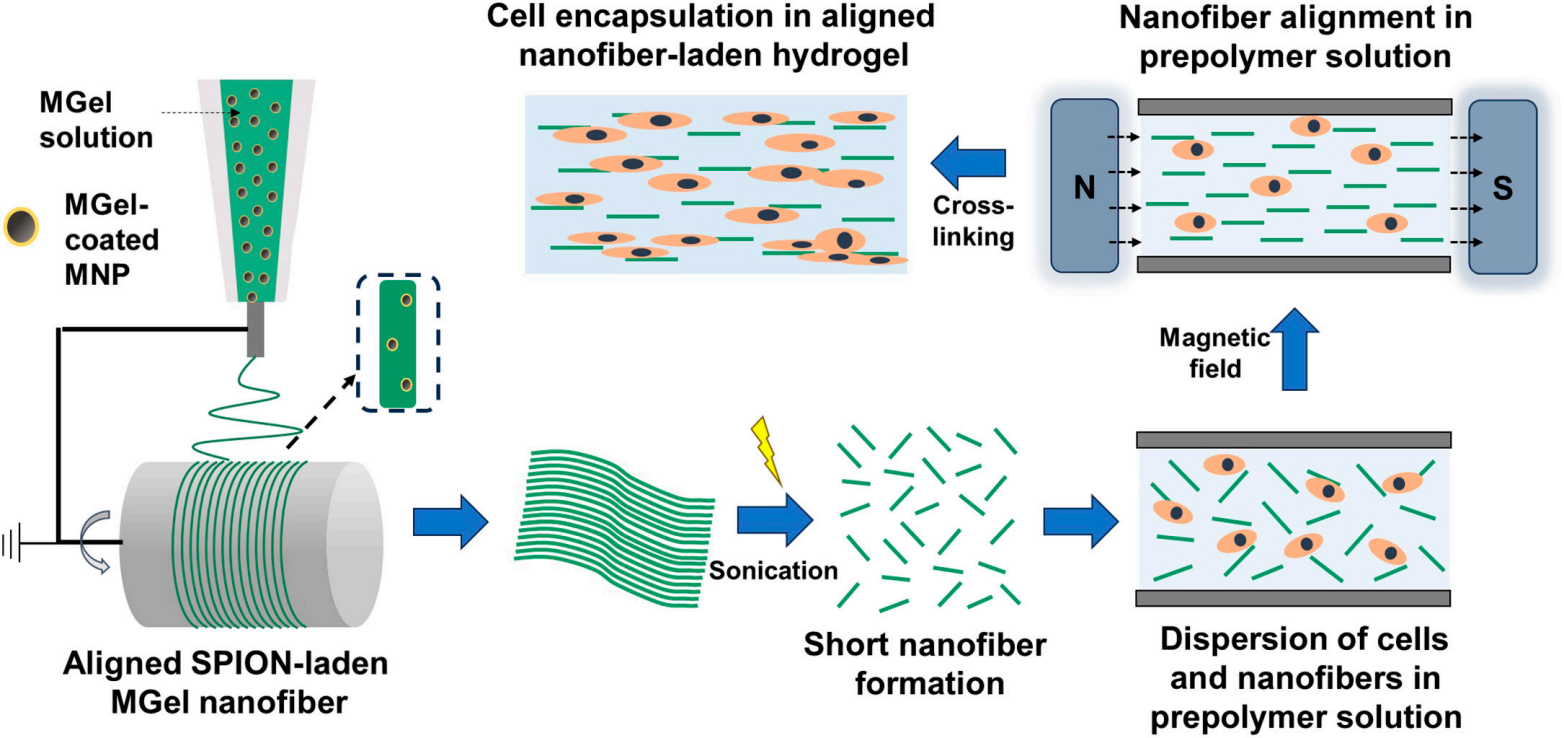
Schematic illustration of the fabrication of aligned nanofiber-laden hydrogel. The aligned magnetic nanoparticle (MNP)-laden nanofiber mat was first created by electrospinning on a rotating drum collector, which was then cut into micrometer-length nanofibers. The short nanofibers and cells were dispersed in a gel prepolymer solution, and external magnetic field was applied to induce the nanofiber alignment within the solution, after which the solution was photocrosslinked in situ to generate the cell-encapsulated, aligned nanofiber-laden hydrogel.

**Fig. 2.**
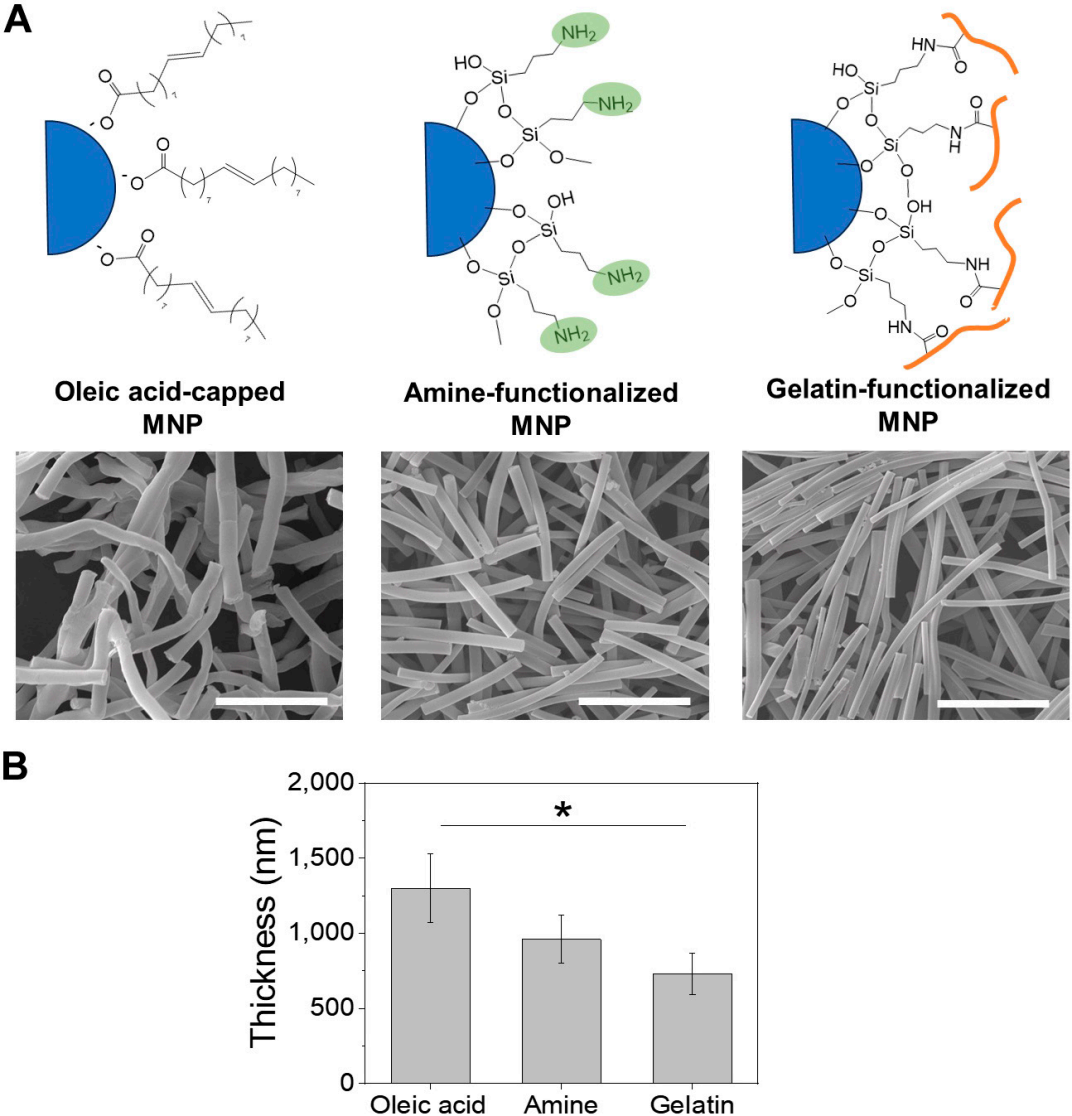
(A) As-fabricated oleic acid-capped MNP was first functionalized with amine via silanization and then conjugated with gelatin via EDC coupling. Scanning electron microscopic (SEM) images of gelatin nanofibers containing oleic acid-capped MNP, amine-functionalized MNP, and gelatin-functionalized MNP (scale bar: 10 μm). (B) The average thickness of the nanofibers incorporating the functionalized MNPs (*P < 0.05, *n* = 10).

Superparamagnetic iron oxide nanoparticles are the most widely used MNPs for biomedical applications for their biocompatibility and tunable surface properties, in addition to magnetic properties [45]. Taking advantage of these attractive qualities of MNPs, the alignment of gelatin nanofibers within a hydrogel was accomplished by incorporating MNP into the nanofibers. The MNP-laden gelatin nanofibers were conveniently developed by electrospinning of gelatin-MNP precursor solution (Fig. 1). While keeping the gelatin concentration constant, MNP content was varied, 0.75, 1.5, 3, and 6 wt%, in order to determine the optimal amount of MNP for magnetic-induced alignment. A relatively small size of the MNP was used in this study, sub-10 nm in diameter, so that a sufficient amount of MNP could be incorporated into nanofibers. In addition, the surface of as-fabricated oleic acid-capped MNP was modified with gelatin via EDC coupling of amine-functionalized MNP in order to stably incorporate into gelatin nanofibers (Fig. 2A). Furthermore, since as-fabricated MNP-laden nanofibers became readily dissolved in aqueous media due to the aqueous solubility of gelatin, the MNP-laden nanofibers were chemically crosslinked via EDC chemistry to impart aqueous stability. The thickness of the MNP-laden nanofibers decreased with gelatin functionalization of MNP (Fig. 2B). This is likely due to the ability of gelatin on MNP to more closely bind to the gelatin nanofiber by both physical interaction and chemical crosslinking, demonstrating the advantage of modifying the MNP surface with gelatin.

**Fig. 3.**
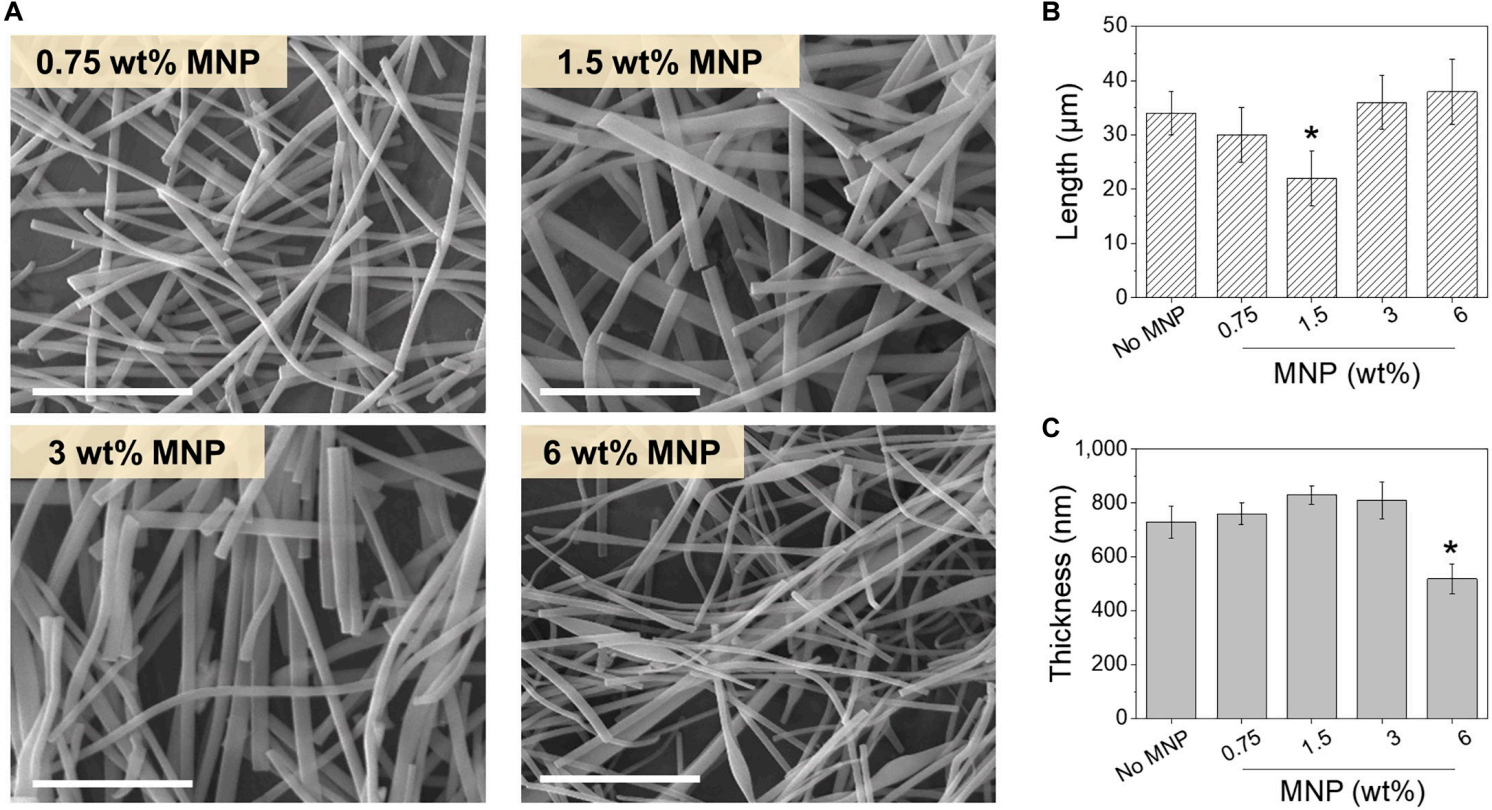
(A) SEM images of MNP-laden gelatin nanofibers cut by ultrasonication (scale bar: 10 μm). The average (B) length and (C) thickness of the short MNP-laden nanofibers (*P < 0.05 when compared with all other conditions, *n* = 10).

MNP-laden nanofibers were treated with ultrasonication to cut them down to micrometer length having aqueous dispersibility (Fig. 3A). The length and thickness of aqueous-dispersible nanofibers were dependent on the MNP content, demonstrating opposite trends. The length showed a biphasic dependence on MNP content, in which the length decreased when MNP content increased from 0.75% to 1.5% but increased when MNP content increased from 1.5 wt% to 6 wt% Fig. 3A and B. On the other hand, the thickness showed the opposite biphasic trend, in which the thickness increased from 0.75% to 1.5% but decreased when MNP content increased from 1.5 wt% to 6 wt% Fig. 3A and C. These interesting results likely stemmed from the composite effect between MNP and the surrounding gelatin matrix [46,47]. At lower MNP concentration, polymer–polymer interaction during the fiber formation far outweighed the MNP–polymer composite formation in overall fiber formation. As a result, the presence of MNP became a critical factor hindering more cohesive fiber formation, as identified by the increase in fiber thickness. In addition, the diminished mechanical strength likely led to shorter length fiber by external force. However, with a further increase in MNP content, the MNP–polymer composite formation had a more prominent effect on the overall nanofiber mechanics, resulting in more cohesive and compact fiber formation. Moreover, the fibers with increased strength were more resistant to external force leading to longer fiber length.

### Magnetic alignment of MNP-gelatin nanofibers in hydrogels

**Fig. 4.**
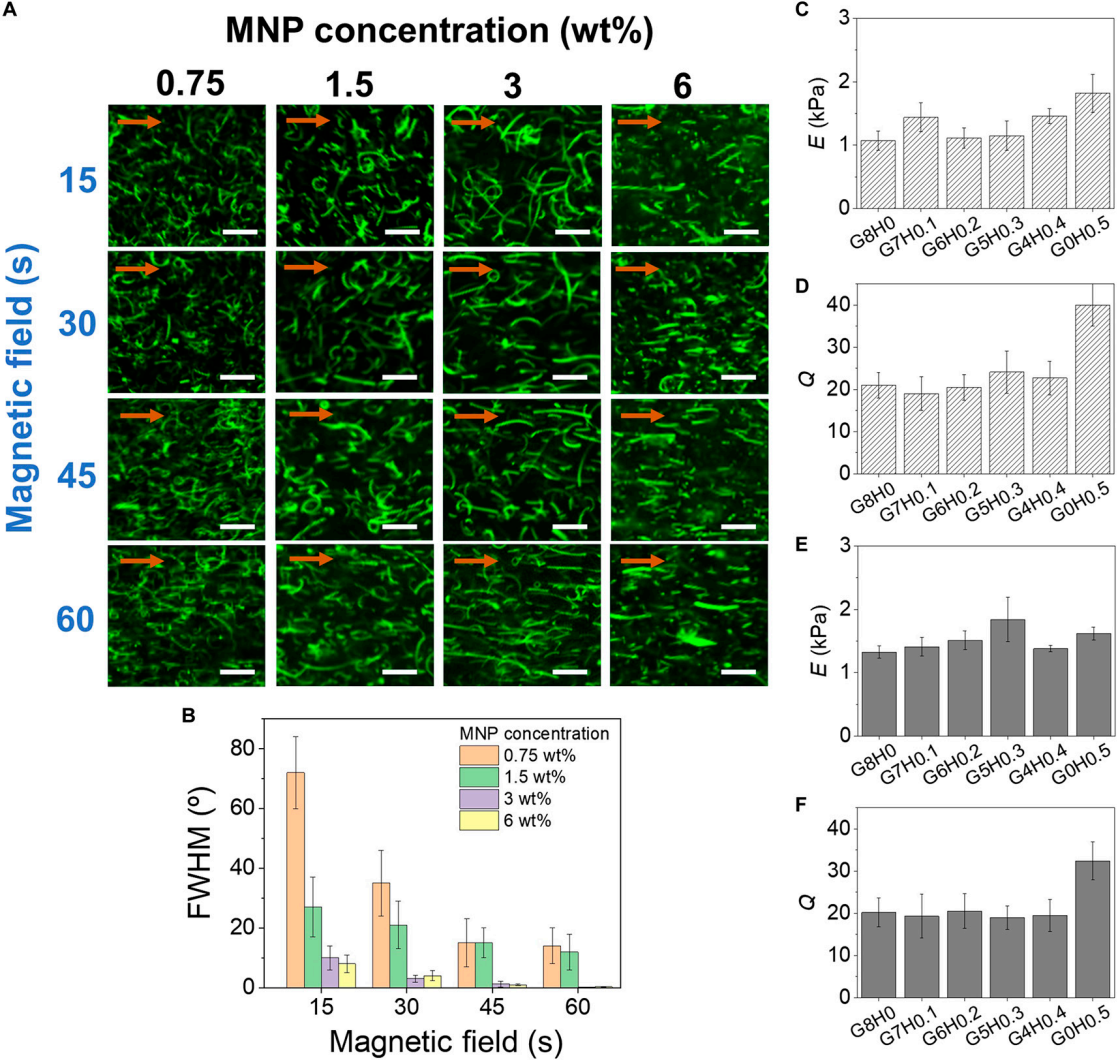
(A) Fluorescent microscopic images of nanofibers dispersed within prepolymer solution subjected to external magnetic field over time (scale bar: 10 μm). The arrows indicate the direction of the magnetic field. (B) The full width at half maximum (FWHM) values of the angular variation of nanofibers from the direction of the magnetic field as the main axis measured at various MNP concentrations and time of magnetic field. Elastic moduli (*E*) and swelling ratios (*Q*) of hydrogels with varying ratios of MGel and MHA (C and D) without nanofibers and (E and F) with nanofibers.

The MNP-gelatin nanofibers dispersed in MGel prepolymer solution were subjected to an external magnetic field to induce their alignment prior to hydrogel fabrication (Fig. 4 and Movie S1). First, the effect of MNP content in nanofiber on the degree of alignment was explored at a given nanofiber concentration at 0.5% (w/v). The rate of fiber alignment increased substantially with MNP content, as expected (Fig. 4A). At lower MNP contents (0.75 wt% and 1.5 wt%), noticeable fiber alignment only began after 30 s, while it only took 15 s or less at higher MNP contents (3 wt% and 6 wt%). Especially at the highest MNP content at 6 wt%, the alignment began almost immediately.

The alignment efficiency was evaluated by the degree of angular variation, which was numerically expressed by FWHM of the histogram of angular variation of nanofibers (Fig. 4B). Decreasing FWHM toward zero meant the majority of nanofibers became aligned along the direction of the magnetic field. It is clearly evident that the FWHM values substantially decreased with MNP content at a given time of magnetic induction. At higher MNP content, the FWHM values became less than 2° after 30 s, demonstrating the supreme efficiency of magnetic-induced alignment.

It should be noted that the external magnetic field applied to induce the alignment could potentially affect the physical properties of the nanofibers by modulating MNP–polymer interaction [48]. To minimize any deleterious change in nanofiber properties, the concentration of MNP was kept low at 3 wt% and the duration of the magnetic field was kept under 1 min for all subsequent experiments.

### Mechanical properties of nanofiber-laden hydrogels

The hydrogels consisting of gelatin and hyaluronic acid were developed by radical copolymerization of methacrylate-functionalized gelation and hyaluronic acid, MGel, and MHA, respectively. Gelatin has been widely used to generate hydrogels for tissue engineering applications, as it is directly derived from collagen, the major component of natural ECM that is largely responsible for mechanical properties of the tissue structures as well as cell adhesive properties [49]. Hyaluronic acid, a natural sulfated polysaccharide that also widely exists in natural ECM, was also incorporated into the hydrogel, since it has been shown to influence a variety of biological activities [50,51]. The concentrations of MGel (“G”) and MHA (“H”) were varied (denoted as “GXHY”, with X and Y representing their concentrations), and their effect on the mechanical properties was first examined by measuring the elastic moduli and swelling ratios.

The overall concentration was kept below 8% (w/v) and the concentration ranges were carefully chosen not to critically alter the mechanical properties, since the objective was to explore the effects of the hydrogel composition and the presence of aligned nanofibers on the encapsulated cell. Any drastic change in mechanics could hamper the independent investigation of the effect of hydrogel composition on cell behavior. From the hydrogel only made of MGel (G8H0), increasing the MHA content up to 0.4% (w/v) while decreasing MGel down to 4% (w/v) (G4H0.4), the mechanical stiffness and permeability were highly consistent, indicating that a similar degree of crosslinking was achieved (Fig. 4C and D). The stiffness showed a meaningful increase only when MHA was increased up to 0.5% (w/v) without MGel (G0H0.5). These results indicated that MHA was mechanically stronger that MGel at a given concentration, so less amount of MHA than MGel was needed to achieve the same mechanical properties. It is interesting to note that despite having higher stiffness, G0H0.5 showed a higher swelling ratio. This was possibly due to the higher hydrophilic nature of hyaluronic acid over gelatin promoting water uptake. As a control group, the hydrogels only made with MGel at different concentrations (G10H0 and G12H0) were also prepared, which predictably demonstrated increasing mechanical stiffness and decreasing swelling ratio with MGel concentration (Fig. S2).

The concentration of MNP-nanofiber infused within the hydrogel was kept low at 0.5% (w/v) to minimize the change in the hydrogel mechanics in order to independently investigate the effect of nanofiber alignment on cell behavior. The stiffness and swelling ratios of the nanofiber-laden hydrogels clearly showed that the mechanical properties of hydrogels were not significantly changed by the presence of MNP-nanofibers, which was likely not high enough to impart reinforcing effect of the composite structure (Fig. 4E and F and Fig. S2).

### Cell morphology in nanofiber-aligned hydrogels

To address the ability of nanofibers aligned within hydrogel to induce the morphological change of encapsulated cells, dermal fibroblasts cultured in gelatin hydrogels embedded with aligned MNP-gelatin nanofibers were studied. First, the biocompatibility of the nanofiber-laden hydrogels and external magnetic field was determined by measuring the viability of the encapsulated cells (Fig. S3). Regardless of the presence of MNP in nanofibers and the application of external magnetic field, their viability was well maintained, all above 85%, demonstrating that the process of magnetic-induced alignment of nanofibers in hydrogel was highly biocompatible. Furthermore, the magnetic field was the same for all conditions; any potential change in nanofiber properties and cells by the magnetic field would be equally applied.

**Fig. 5.**
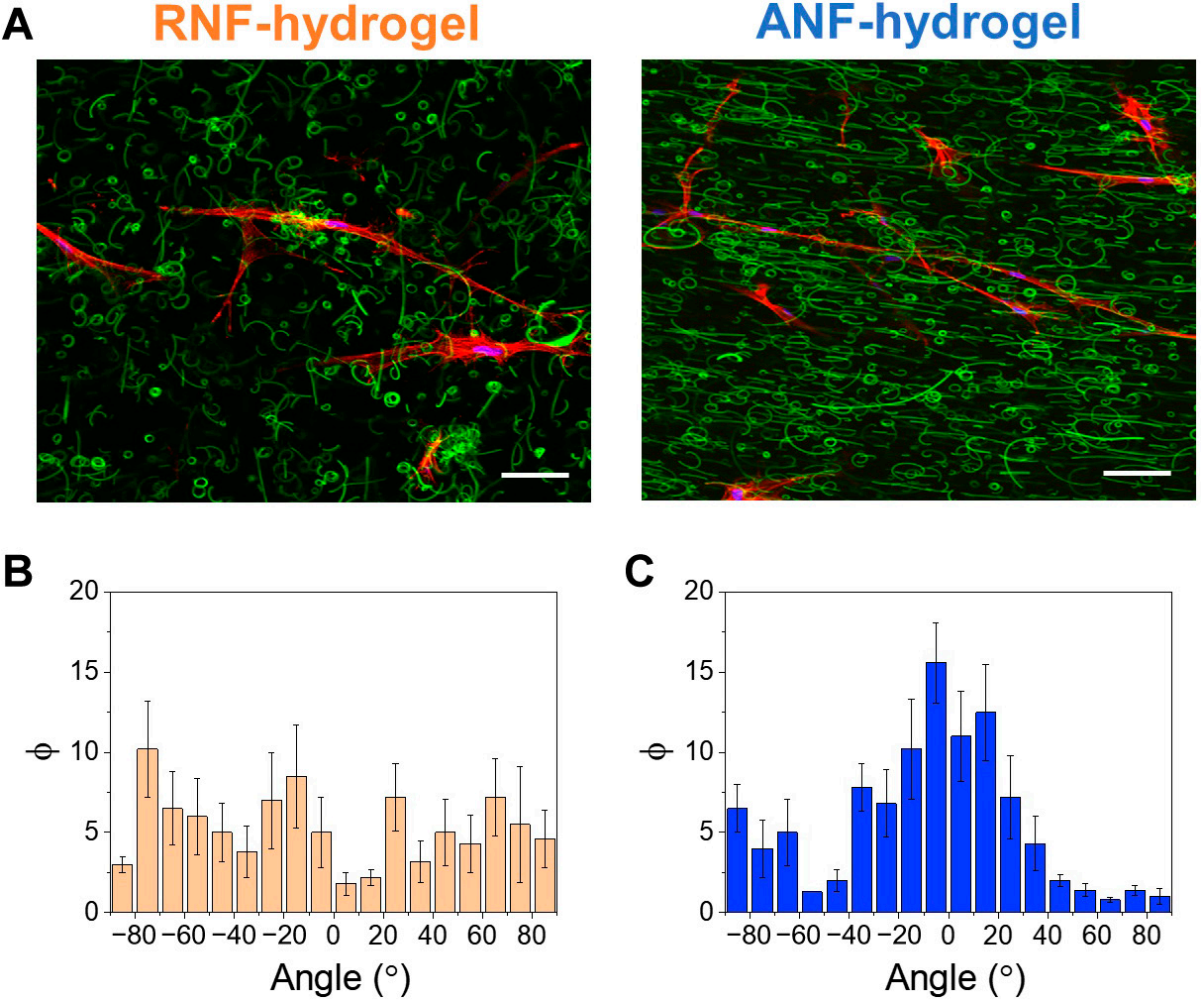
(A) Representative fluorescent microscopic images of fibroblasts in gelatin hydrogels with randomized nanofibers (RNF-hydrogel) and aligned nanofibers (ANF-hydrogel) (scale bar: 10 μm). The percentages of elongated cells (φ) oriented different angles from the randomly chosen axis for (B) RNF-hydrogel and from the axis of aligned nanofibers in (C) ANF-hydrogel.

Then, the cells were encapsulated in hydrogels having randomly dispersed nanofibers (“RNF-hydrogel”) without magnetic field or aligned nanofibers (“ANF-hydrogel”) with magnetic field and cultured over time, and the directional elongation along the nanofiber orientation was evaluated (Fig. 5). The cells in RNF-hydrogel did not demonstrate any preferential direction of elongation, while a considerable portion of the cells in ANF-hydrogel became elongated along the direction of the aligned nanofibers. The cells in RNF-hydrogel showed a high degree of cell spreading and elongation, albeit to a lesser extent than those in ANF-hydrogel. This was similarly demonstrated in a previous study in which increased surface roughness imparted by the presence of nanofibers significantly contributed to increased cell adhesions and elongation (Fig. 5A and B) [15]. However, the elongated cells did not preferentially orient themselves in any particular direction in RNF-hydrogels. On the other hand, the elongated cells in ANF-hydrogel not only showed a much larger aspect ratio, but also demonstrated preferential orientation along the direction of aligned nanofibers (Fig. 5A and C).

The fibroblasts were cultured on 2D surface coated with random nanofibers and aligned nanofibers to highlight the difference in spatial distribution of nanofibers with respect to the cells (Fig. S4). The nanofibers on surface would only contact a small area of the cell, while those within hydrogel would surround the entire cell area. Similar to the nanofiber-laden hydrogel, the cells also became more elongated along the aligned nanofibers, while those on random nanofibers showed more radial spreading on the surface. However, the cell aspect ratio on the aligned nanofibers was not as high, because the cells, which are much larger than the nanofibers on the surface, could more easily cross over to surrounding area with nanofibers. This result further underscored the dominant effect of aligned nanofibers in 3D hydrogel environment on guiding the anisotropic cell morphology by restricting their motility.

#### Effect of ECM composition

Our previous study has clearly indicated that the presence of nanofibers within gelatin hydrogel promoted the spreading and proliferation of dermal fibroblasts through increased mechanical stiffness and surface roughness, providing additional adhesion cites for cells [15]. Herein, the composition of hydrogel was varied by incorporating hyaluronic acid into gelatin hydrogel in addition to inducing the alignment of nanofibers, since hyaluronic acid has been implicated in modulating wound healing and fibrotic activities of fibroblasts differently from gelatin [50–57].

**Fig. 6.**
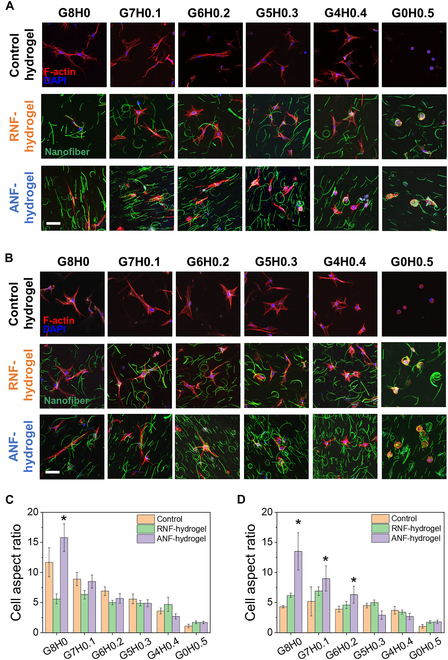
(A and B) Representative fluorescent microscopic images of dermal fibroblasts in control hydrogels (without NF), RNF-hydrogels, and ANF-hydrogels with varying MHA concentrations (scale bar: 10 μm); (A) normal culture, (B) supplemented with TGF-β. F-actin and nuclei of the cells and the nanofibers were fluorescently labeled. The cell aspect ratios were quantified by measuring the maximum and minimum lengths of cells in (A) and (B) and presented in (C) and (D), respectively (*P < 0.05, when compared with other nanofiber conditions in the same hydrogel, *n* = 10).

For control hydrogels without nanofibers, the fibroblasts encapsulated in hydrogels showed extensive spreading in all compositions (Fig. 6A and C). For pure MGel hydrogel (G8H0), the cells became highly elongated (“fibroblastic”) rather than more evenly stretched (“epithelial-like”), which is commonly demonstrated in a collagen-rich and mechanically stiff environment [58]. This polarity is often associated with high motility and proliferative capacity. However, increasing MHA content, especially at the highest MHA (G4H0.4), significantly reduced the cell aspect ratio, with the cells acquiring more epithelial-like morphology. This change in morphology strongly suggested reduced motility due to both increased MHA and decreased MGel. This result is in line with the previous studies demonstrating reduced migration and collagen synthesis of fibroblasts by binding with hyaluronic acid [53,59].

For RNF-hydrogels, extensive cell elongation that was evident for pure hydrogels was not observed even at low MHA content (Fig. 6A and C). It is likely that the presence of nanofibers randomly distributed within the hydrogel blocked the cells from more extensive elongation, but more importantly, the cells were more epithelial-like, demonstrating more lamellipodial projections. Given these lamellipodia were extended toward the nanofiber, it suggested that gelatin nanofibers provided more cites for cell adhesions. The decrease in the cell aspect ratio with increasing MHA concentration was also observed for RNF-hydrogel, further demonstrating the potent biophysical effect of HA on diminishing the motile behavior of the fibroblasts.

The overall effect of ANF-hydrogels on the encapsulated fibroblasts was not dissimilar to control hydrogels and RNF-hydrogels (Fig. 6A and C). The biggest difference was the much larger cell aspect ratio for ANF-hydrogels at lower MHA concentrations. This result, along with Fig. 5, proved that the aligned nanofibers helped guide the cells to further elongation. Even with the greater cell elongation provided by the aligned nanofiber, the critical effect of MHA on reducing the cell elongation was also on display, reducing down to aspect ratios similar to those of control hydrogels and RNF-hydrogels with increasing MHA concentration.

**Fig. 7.**
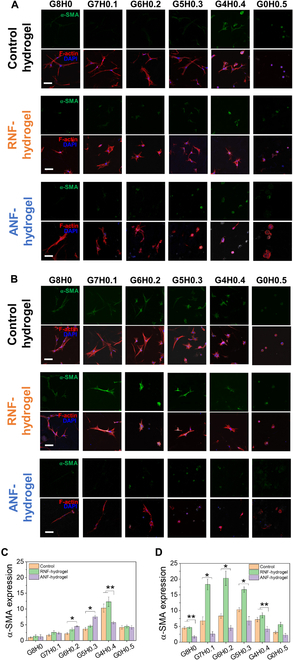
Representative fluorescent microscopic images of fibroblasts cultured in control hydrogel (without NF), RNF-hydrogels, and ANF-hydrogels with varying MHA concentrations (scale bar: 10 μm); (A) normal culture, (B) supplemented with TGF-β. Alpha smooth muscle actin (α-SMA) was fluorescently labeled (green). F-actin (red) and nuclei (blue) were also fluorescently labeled to visualize the overall cell morphology. (C and D) The amounts of α-SMA expression per cell shown in (A) and (B) were quantified by the overall intensities of fluorescent signals (*P < 0.05 when compared among 3 conditions, **P < 0.05 when compared with 2 conditions, *n* = 10). The values were normalized with that for G8H0 of control hydrogel.

Fibroblasts undergo differentiation into myofibroblasts that are responsible for increased fibrosis during wound healing, as identified by increased cell spreading, motility, ECM production, and tissue contractility [60]. The increased cell spreading and elongation suggested that the fibroblasts in RNF-hydrogels and ANF-hydrogels may have acquired the myofibroblastic phenotype. Therefore, the expression of α-SMA, a hallmark of myofibroblasts, was measured via immunocytochemistry to analyze the myofibroblast differentiation in detail (Fig. 7).

Regardless of the presence and alignment of nanofibers in hydrogels, α-SMA expression was minimal in most of the conditions, suggesting that the increasing cell elongation did not necessarily correlate with and was indicative of the myofibroblastic differentiation (Fig. 7A and C and Fig. S5A). The only notable increase in α-SMA expression was demonstrated for G4H0.4 for all nanofiber conditions, which suggested that the increasing presence of HA had a small but meaningful impact on the myofibroblastic differentiation. It was speculated that rather than an extremely high elongation, a certain combination of fibroblastic and epithelial-like morphologies was more favorable for myofibroblastic differentiation. Additionally, since G0H0.5 did not further enhance the α-SMA expression and rather showed decreased expression, the cell adhesion and spreading provided by gelatin was a crucial prerequisite.

#### Effect of soluble factor (TGF-β)

The myofibroblastic differentiation and fibrotic activity of fibroblasts are controlled by a host of soluble factors, including TGF-β, which has been shown to play a prominent role [61]. In order to further explore the effects of aligned nanofibers and the presence of HA within gelatin hydrogel on the fibroblasts, the cells encapsulated in the hydrogels were treated with TGF-β and the cell aspect ratio and α-SMA expression were analyzed.

There was a significant increase in the α-SMA expression and decrease in the cell aspect ratios in all hydrogel compositions for control hydrogels (Figs. 6B and D and 7B and D and Fig. S5B). Similarly, the α-SMA expression also showed marked increase for RNF-hydrogels, even higher than control hydrogels, while the cell aspect ratios remained low and similar to those of control hydrogel. For both hydrogels, the α-SMA expression increased with MHA concentration, recording the maxima at G6H0.2 and G5H0.3, but tapered off at higher MHA concentration. This result confirmed the synergistic role of the soluble factor and the ECM component on modulating the myofibroblastic differentiation.

The possibility of high cell aspect ratio opposing myofibroblastic differentiation was further corroborated by the observation that the α-SMA expression of the cells in ANF-hydrogels was kept low regardless of MHA concentration even under the influence of TGF-β. Since α-SMA expression was shown to be enhanced by TGF-β in the presence of randomly dispersed nanofibers, the cells that were induced to display elongated morphology by the aligned nanofiber could not undergo myofibroblastic differentiation, even with TGF-β and MHA.

To better highlight the results obtained from the hydrogels containing both gelatin and hyaluronic acid at similar mechanical rigidity, the fibroblasts in the control group of pure MGel hydrogels at different MGel concentrations (G8H0, G10H0 and G12H0) was explored. The cell elongation decreased with increasing MGel concentration most likely due to the limited inner space within the hydrogel at the higher degree of crosslinking (Fig. S6). The effect of randomized or aligned nanofibers within the hydrogel on the cell elongation was similar to that of MGel-MHA hydrogels, where the cell aspect ratios in ANF-hydrogels were higher than those in RNF-hydrogels regardless of the presence of TGF-β. More importantly, the α-SMA expression remained low at all hydrogel compositions and only a small increase was induced by TGF-β (Fig. S7). Since the cell aspect ratios were similar, it could be inferred that MHA played a crucial role in mediating the myofibroblastic differentiation through not only controlling the cell morphology more conducive to cell spreading, but also biophysical signaling via specific cell–hyaluronic acid binding.

### In vivo evaluation of bioactivity of nanofiber-laden hydrogels

**Fig. 8.**
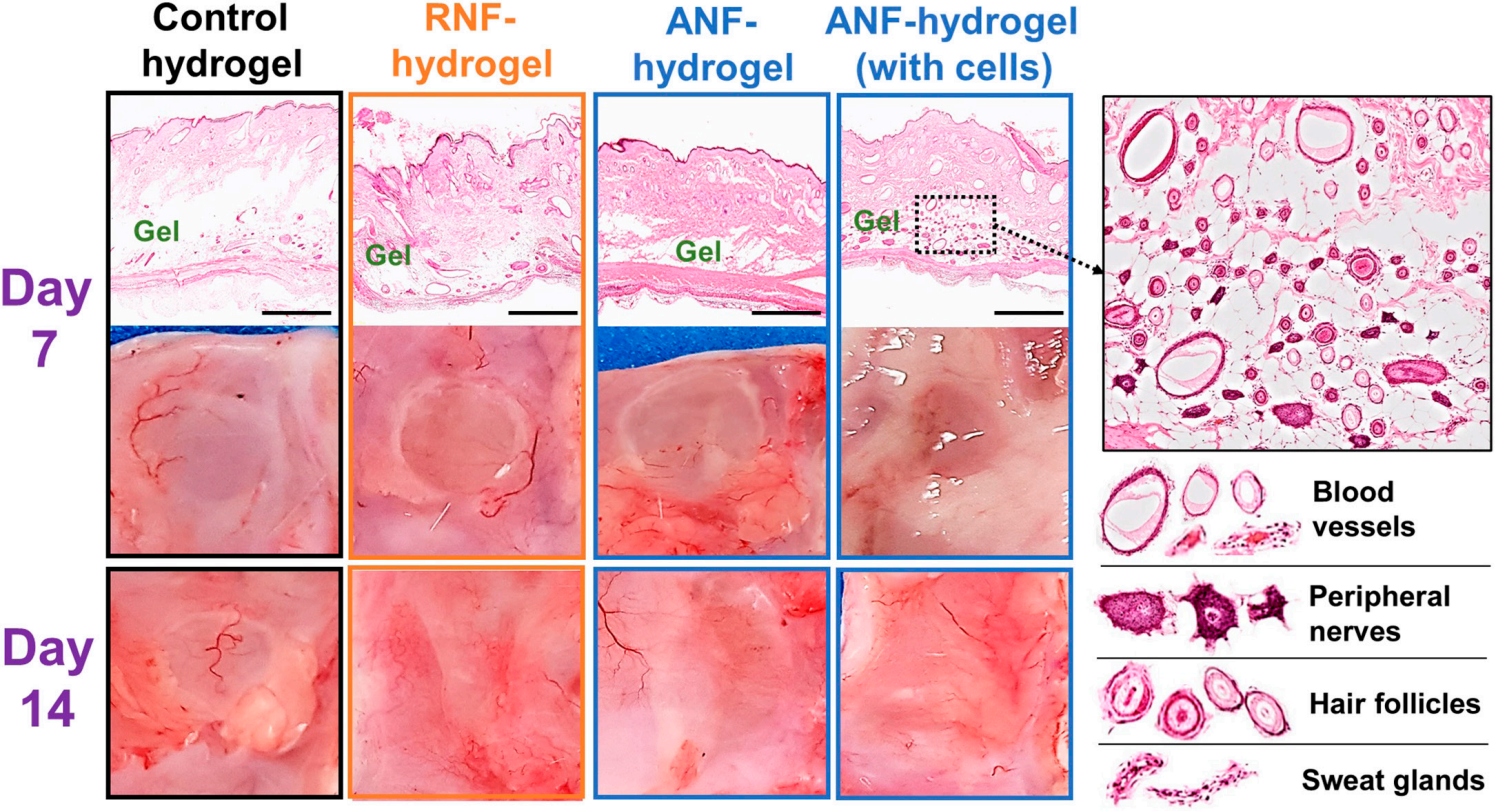
Photographs and histological microscopic images of hydrogel (without nanofiber or cell), RNF-hydrogel, ANF-hydrogel, and ANF-hydrogel with encapsulated fibroblasts subcutaneously implanted in mice (scale bar: 1 mm). A magnified image of ANF-hydrogel with fibroblasts are shown on the right, showing various tissue structures of the skin infiltrated into the hydrogel.

In addition to the in vitro evaluation of various phenotypes of encapsulated dermal fibroblasts, the nanofiber-laden hydrogels were further evaluated for their ability to function as implantable engineered tissues. The control hydrogel without embedded nanofibers, RNF-hydrogel, ANF-hydrogel, and fibroblast-encapsulated ANF-hydrogel were subcutaneously implanted in SD rats up to 14 days, and the hydrogel and the surrounding tissue were histologically analyzed (Fig. 8). After 7 days, it was clearly evident from the photographs that the surrounding blood vessels extensively infiltrated into all hydrogels. More detailed histological images clearly demonstrated the angiogenesis within the hydrogel, highlighting the biocompatibility and the regenerative capacity of the hydrogel consisting of gelatin and hyaluronic acid and the gelatin nanofibers. This observation was in line with several previous studies demonstrating the angiogenic effect of gelatin and hyaluronic acid even without soluble factors or cells due to the abundance of integrin-binding domains in gelatin and hyaluronic acid recruiting various cells for tissue growth [62–65].

More importantly, ANF-hydrogel encapsulating fibroblasts induced much more extensive ingrowth of different types of specialized tissue structures, including peripheral nerves, hair follicles, and sweat glands, in addition to blood vessels, while other conditions devoid of fibroblasts displayed such tissues in much lesser extent. This remarkable result certainly highlighted the role of aligned fibroblasts exerting promoting the skin regeneration and tissue ingrowth into the hydrogel.

It should also be pointed out that there was no evidence of marked presence of inflammatory cells, such as macrophages and lymphocytes, within the vicinity or inside the hydrogels. Furthermore, no visible evidence of organ toxicity, especially in liver and kidney, was observed. These results evidently proved the biocompatibility of the nanofiber-laden hydrogels.

The nanofiber-laden hydrogels also demonstrated biodegradability. After 14 days, all the nanofiber-laden hydrogels fully degraded, while for the control hydrogel, a substantial portion still remained. The ANF-hydrogels with fibroblasts showed the fastest degradation rate, followed by ANF-hydrogel and RNF-hydrogel. The rate of degradation closely followed that of skin tissue ingrowth, which indicated that the degradation was facilitated by the cellular activities.

### Multi-layered heterostructure hydrogel with differential nanofiber alignments

**Fig. 9.**
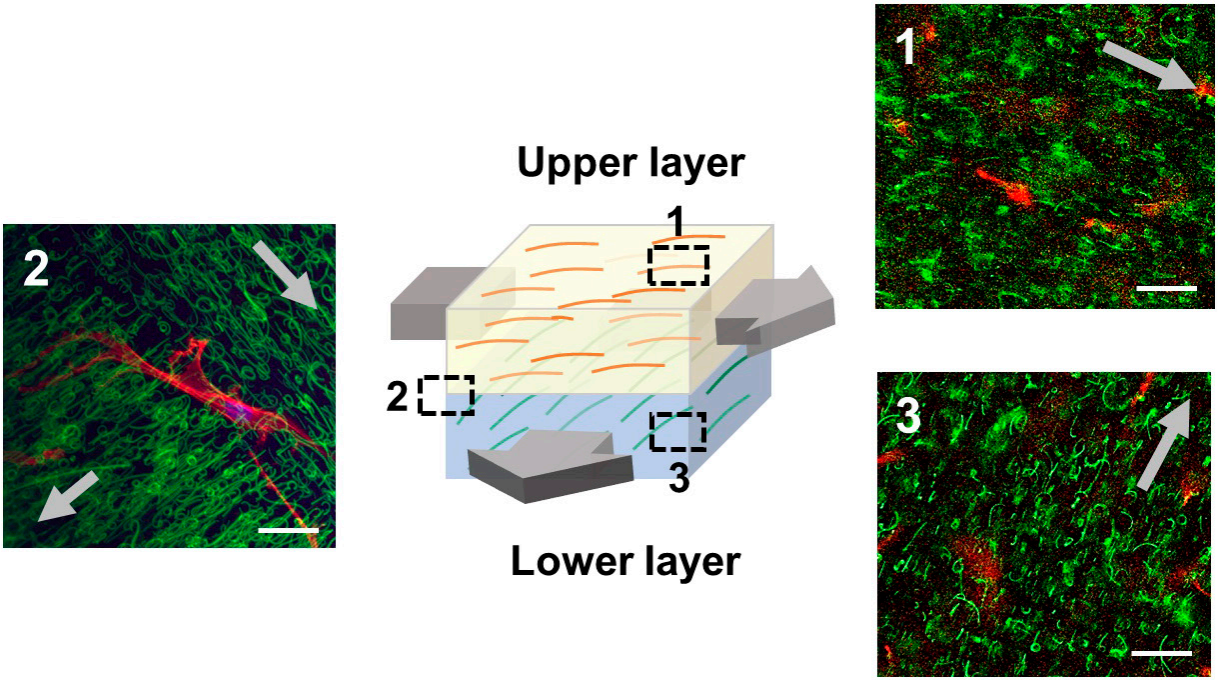
Schematic illustration of a multi-layer heterostructure consisting of hydrogels with different nanofiber alignments and the fluorescent microscopic images of fibroblasts encapsulated in the hydrogel layers with different nanofiber alignments and at their junction (scale bar: 20 μm). F-actin (red) of the cells and the nanofibers (green) were fluorescently labeled. The arrows indicate the direction of the magnetic field.

Since the nanofiber alignment can be efficiently accomplished by external magnetic field applied to the prepolymer solution, which can be photocrosslinked in situ to form the nanofiber-composite hydrogel, this process can be performed repeatedly to generate a larger and more complex, anisotropic nanocomposite hydrogel consisting of compartmentalized hydrogels having different alignments of nanofibers. To demonstrate this versatility, a hydrogel consisting of 2 nanofiber-composite hydrogel layers having different directions of nanofiber alignments was developed via layer-by-layer fabrication. A hydrogel layer consisting of aligned nanofibers was first created, which was immediately followed by placing a new prepolymer solution, applying the magnetic field in the perpendicular direction to the first nanofiber alignment, and photocrosslinking to generate the second hydrogel layer (Fig. 9 and Movie S2). The fibroblasts cultured in these 2 layers showed similar directional elongations along the respective nanofiber alignments. Furthermore, the cell located at the interface of these 2 layers could cross over to both sides and elongate in perpendicular directions, which clearly demonstrated that the hydrogel layers were well integrated, and the cells could sense the changing environment.

## Discussion

The study demonstrated that the nanofibers embedded inside the hydrogel helped guide the cells to acquire particular morphologies based on the nanofiber orientation, much like the natural collagen fibrils. The crucial feature of the nanofibers to impart this effect was the cell adhesive properties of the gelatin nanofibers. It could be argued that the presence of nanofibers simply may have provided a physical barrier to prevent from spreading to other areas. However, if the nanofibers were not cell adhesive, the cells could have preferentially and more freely migrated and spread through the area of hydrogel without nanofibers. Since the cells were observed to adhere to the nanofibers, they acquired highly elongated morphology along the aligned nanofibers or epithelial-like morphology bound to randomized nanofibers.

In addition to controlling the orientation of gelatin nanofibers, providing hyaluronic acid was another crucial design principle to control the fibroblast activities. While gelatin, a derivative of collagen, provides adhesion sites, hyaluronic acid has also been implicated in modulating various activities of dermal fibroblasts via CD44 and receptor for hyaluronic acid-mediated motility [51,52]. For example, hyaluronic acid has been shown to increase the cell spreading and proliferation of dermal fibroblasts via increasing the cell cycle with tubulin biosynthesis [50,53]. In addition, hyaluronic acid also mediates wound healing, in which high-molecular-weight hyaluronic acid deposited into ECM reduces scarring by limiting collagen biosynthesis and angiogenesis, while the enzymatic breakdown of high-molecular-weight hyaluronic acid into lower-molecular-weight fragments facilitates inflammatory response [54–57].

Surprisingly, the reduction in cell spreading within the hydrogel with increasing hyaluronic acid reached a point where the fibroblasts in pure hyaluronic acid hydrogel (G0H0.5) did not display any meaningful cell spreading. Without cell adhesion moieties (e.g., RGD peptides) provided by gelatin, the cells could not effectively display filopodial and lamellipodial protrusions without forming focal adhesions [66]. However, the cells did show spreading along the nanofibers in pure MHA hydrogel, which was a further proof that the gelatin nanofibers provided sites for cell adhesion.

The activation of quiescent fibroblasts to increased proliferation and migration during wound healing is characterized by the formation of α-SMA stress fibers integrated with the F-actin cytoskeleton. The elevated level of α-SMA at the intermediate hyaluronic acid content and nanofibers, along with TGF-β, suggested that the influence of soluble factors, the topological structure of matrix, and the presence of hyaluronic acid could all combine to activate the fibroblasts to become myofibroblasts that are largely responsible for wound contraction and tissue regeneration.

It is interesting to note that an intermediate degree of cell aspect ratio, in which the cells acquired more epithelial-like radial spreading while the extreme directional elongation was attenuated, was more conducive toward myofibroblastic differentiation. One crucial characteristic of myofibroblasts is the migratory behavior, which is manifest in the formation of lamellipodia and filopodia. The cells that have underwent elongation and acquired extremely high aspect ratios likely lost their migratory potential, with the reduced number of lamellipodia and filopodia and the entropically unfavorable morphology, which has been similarly suggested previously [67].

Several studies have pointed out that the specific topological alignment of the matrix structure can considerably change the cytoskeletal tension [68]. Within a randomly organized structure, cells detect radial tension along rigid fibers. Therefore, consistent and robust contractile forces radially exerted upon TGF-β-activated fibroblasts promote the maturation of α-SMA. Conversely, within an aligned structural framework, relatively lower tension is exerted in a singular direction, hindering the α-SMA maturation even under the influence of TGF-β. This result evidently underscores the importance of the physicomechanical properties of cell culture platforms that exert considerable influence over various phenotypes of the cells.

Fibroblasts play a crucial role in skin physiology and wound healing by producing a wide array of growth factors and cytokines [69]. The fibroblasts residing in a physiologically favorable microenvironment provided by hydrogel are able to produce growth factors and cytokines in a more sustained manner to induce angiogenesis and wound healing [70,71]. Therefore, the bioactivity of nanofiber-laden hydrogel, coupled with the paracrine effect of fibroblasts, substantially influenced the regeneration of skin tissue. The ingrowth of specialized structures into the ANF-hydrogel with fibroblasts, which signified the mature skin regeneration, was likely facilitated by the initial formation of extensive blood vessels, which are known to regulate their growth and maturation, often alongside one another [72–74]. The fibroblasts also likely played a meaningful role in this ingrowth, as evidenced by the cells surrounding these structures. The faster degradation of the nanofiber-laden hydrogel was also likely facilitated by the increased activity of fibroblasts producing ECM-degrading enzymes and matrix metalloproteinases [75].

The ultimate goal of tissue engineering is to emulate the complexity of native tissue structures, which is why the multi-layered nanofiber-laden hydrogel having heterogeneous nanofiber alignment has a great potential in the future of tissue engineering. The fabrication process for the nanofiber-laden hydrogel, which entails the magnetic induction of nanofiber alignment in the prepolymer solution followed by photocrosslinking, makes it possible to apply multiple iterations of this process to generate a larger structure having different nanofiber alignments. This study showcased a simple proof-of-concept demonstration of this attribute. However, there are several technical hurdles that need to be overcome in order to achieve more complex heterostructures with greater clinical potential and applicability. First of all, since the nanofiber alignment is accomplished by an external magnetic field, it is quite time-consuming, more so than applying mechanical force, which may be harmful to cells exposed to prolonged magnetic field. In addition, hydrogels with larger dimensions cause diminishing diffusivity of surrounding medium toward the core, which could become detrimental to the viability of the encapsulated cells. It is expected that a more sophisticated and automated system, which can safely streamline this process and precisely control the dimensions to create pores or channels for increased permeability and angiogenesis, is expected to overcome these issues [10,70].

In this study, an innovative 3D cell culture platform was developed by incorporating anisotropically positioned short nanofibers emulating natural collagen fibrils into a hybrid hydrogel consisting of gelatin and hyaluronic acid. The gelatin nanofibers including MNPs could be efficiently aligned within a prepolymer solution by mild external magnetic field, which could be crosslinked in situ to develop the aligned nanofiber-embedded hydrogels. Furthermore, the fabrication process can be repeated while changing the direction of the magnetic field to develop a more complex nanofiber-composite hydrogels with heterogeneous nanofiber alignments. This nanofiber-composite hydrogel was used as a 3D scaffold to culture dermal fibroblasts, as the ability to incorporate and control the physicomechanical properties via nanofiber alignment as well as the hydrogel composition could provide the microenvironment conducive to the fibroblasts. The results strongly indicated that the presence of nanofibers and a certain amount of hyaluronic acid in hydrogel promoted myofibroblastic differentiation, which was further enhanced by TGF-β, a known mediator of fibrosis. It was poignant that the maximal differentiation was demonstrated for the cells acquiring epithelial-like radial cell spreading and diminished directional elongation with increasing hyaluronic acid concentration compared to those with extremely high cell aspect ratio. This fascinating result was further corroborated by the diminished myofibroblastic differentiation of cells having higher aspect ratios in hydrogels with aligned nanofibers, even under the influence of TGF-β. Taken together, the results provided herein demonstrated that the aligned nanofiber-laden gelatin-hyaluronic acid hydrogel that could control both the composition and microstructure could serve as a 3D cell culture platform that can accurately and properly control the complexity of a cellular microenvironment for a wide array of cells and tissues.

## Data Availability

The data used and/or analyzed during the current study are available from the corresponding author on reasonable request.
