# Supplementary material for: Multiscale Control of Nanofiber-Composite Hydrogel for Complex 3D Cell Culture by Extracellular Matrix Composition and Nanofiber Alignment
Source: Biomater Res. 2024 May 29;28:0032. doi: 10.34133/bmr.0032 (PMC11136538; doi:10.34133/bmr.0032)
Supplement: Supplementary 1 — Materials and Methods Figs. S1 to S7 Movies S1 and S2 [file bmr.0032.f1.zip › Figs. S1 to S7.docx]

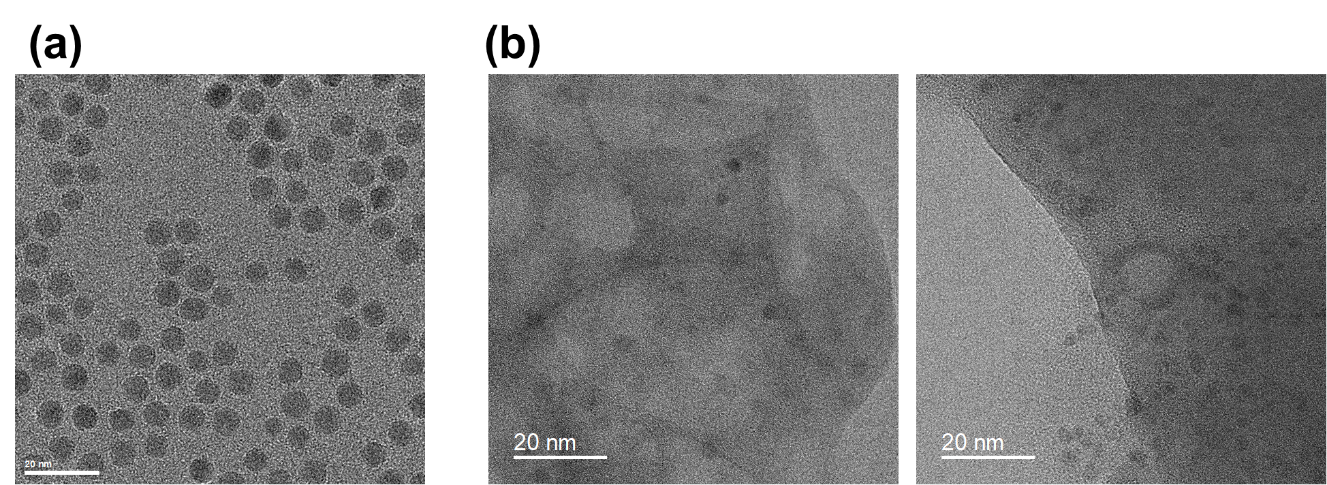


**Figure S1.** Representative transmission electron microscopic images of (a) as-fabricated MNP and (b) MNP-laden gelatin nanofibers.


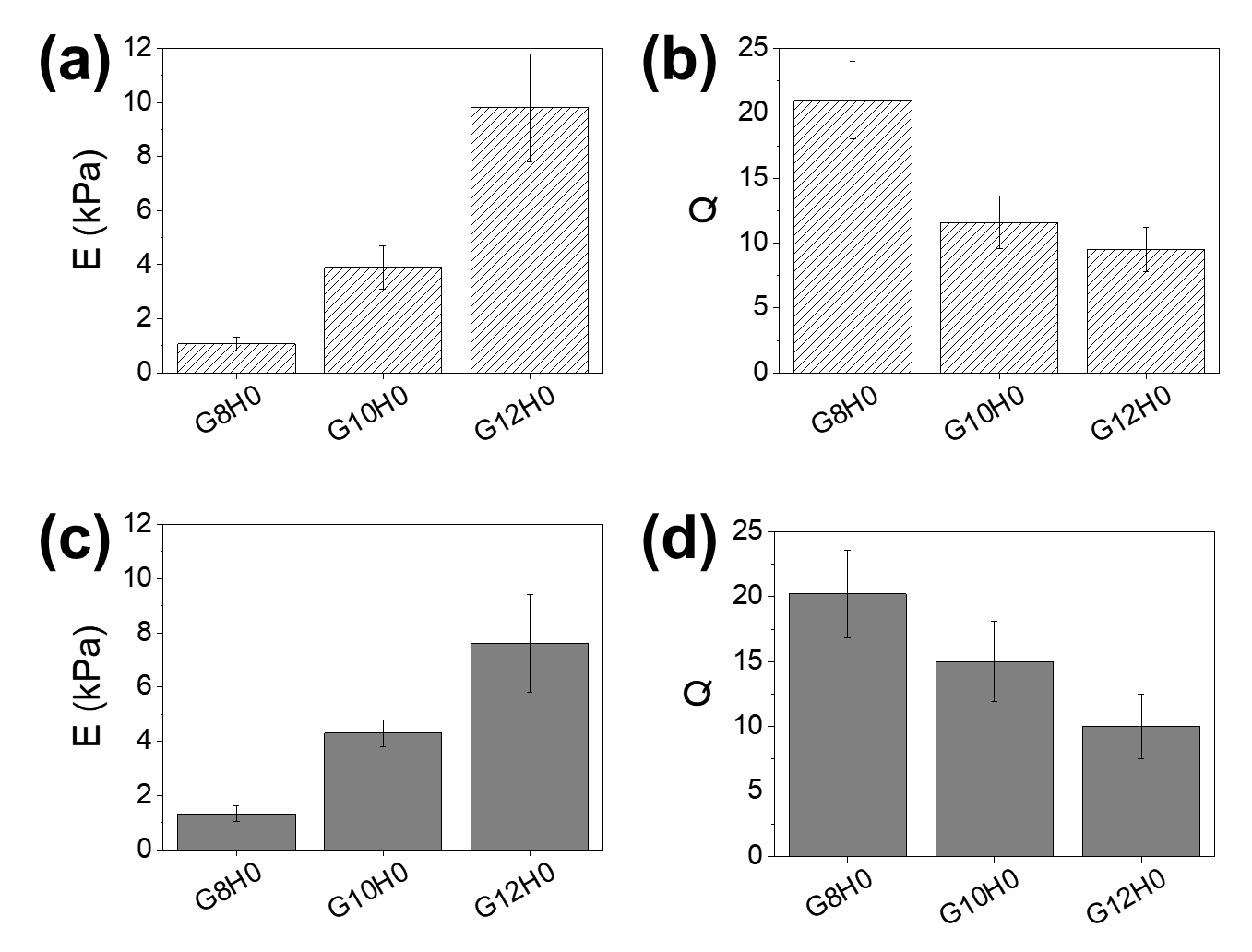


**Figures S2.** Elastic moduli (*E*) and swelling ratios (*Q*) of hydrogels with varying gelatin concentrations; 8 % (G8H0), 10 % (G10H0) and 12 % (G12H0) (a, b) without nanofibers and (c, d) with nanofibers.


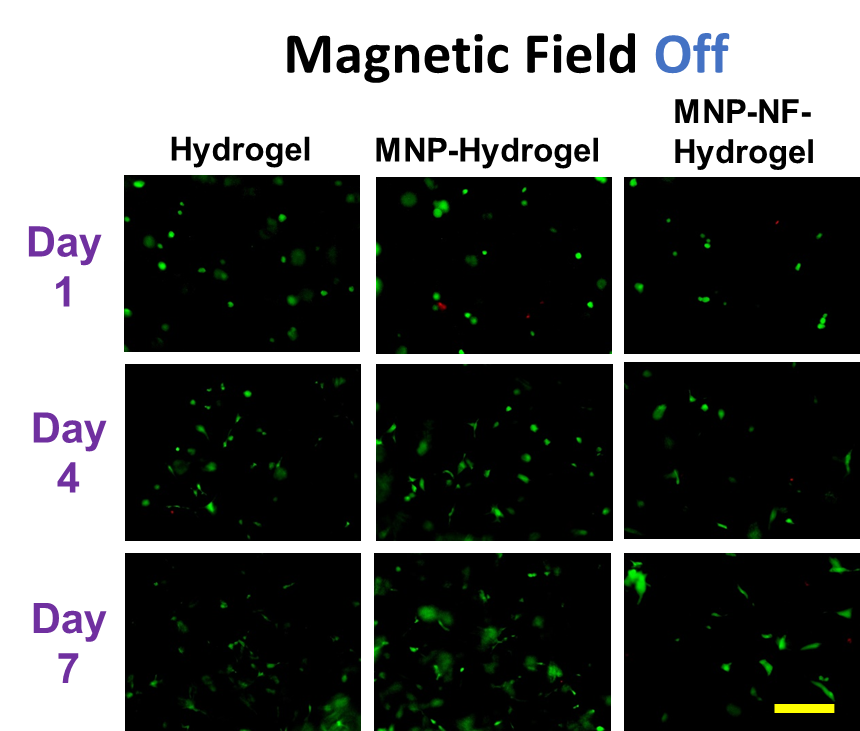


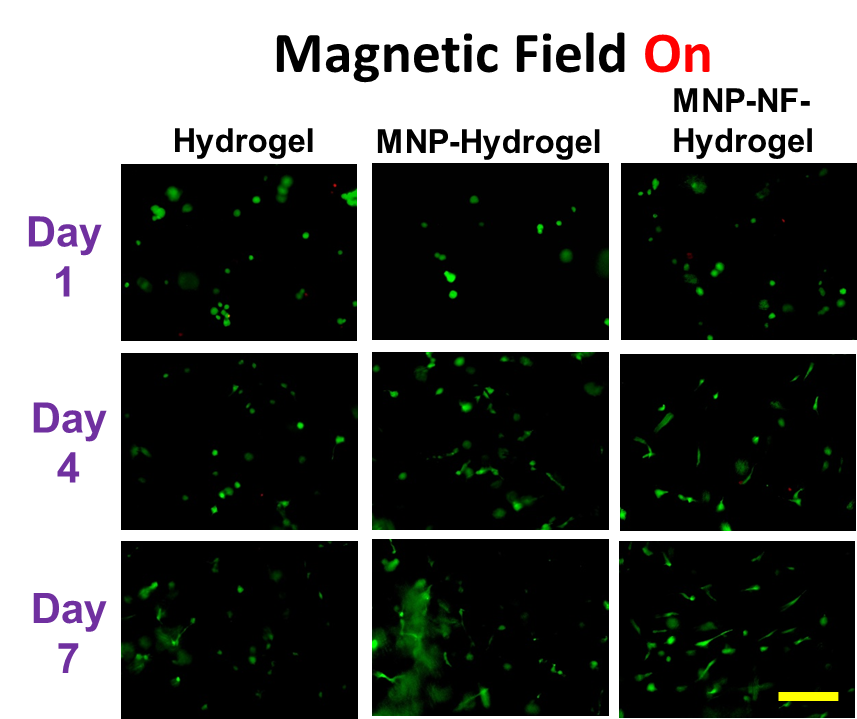


**Figure S3.** The cytotoxicity evaluation was performed by measuring the viability of dermal fibroblasts in gelatin hydrogels embedded with MNP or MNP-laden nanofibers via fluorescently labeling live (green) and dead (red) cells (scale bar: 100 µm).


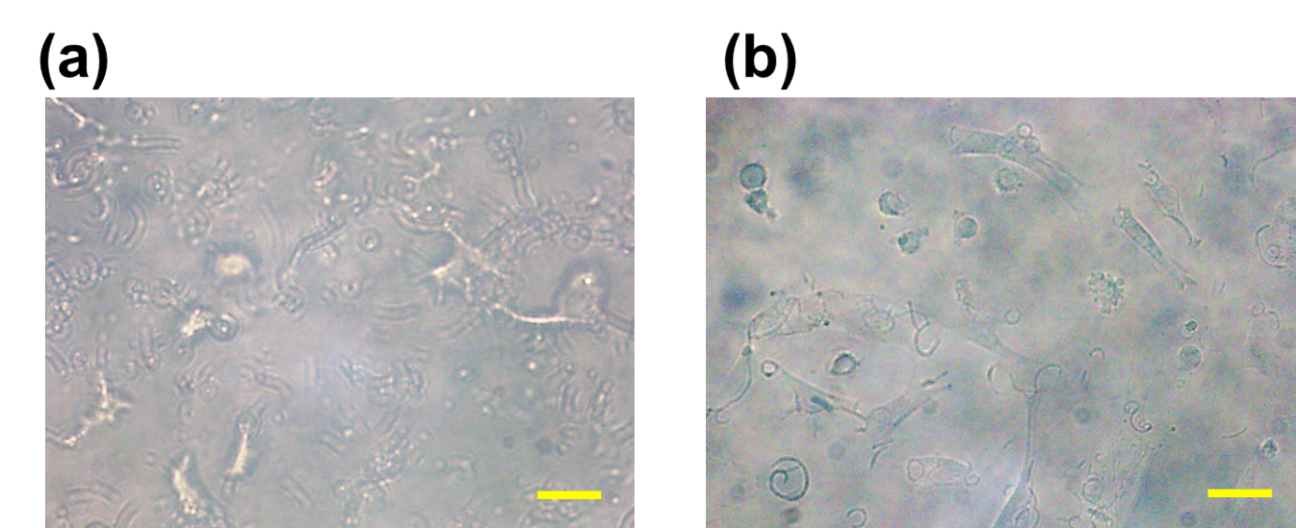


**Figure S4**. Optical microscopic images of dermal fibroblasts cultured on 2D surface coated with (a) random nanofibers and (b) aligned nanofibers one day after seeding (scale bar: 20 µm).


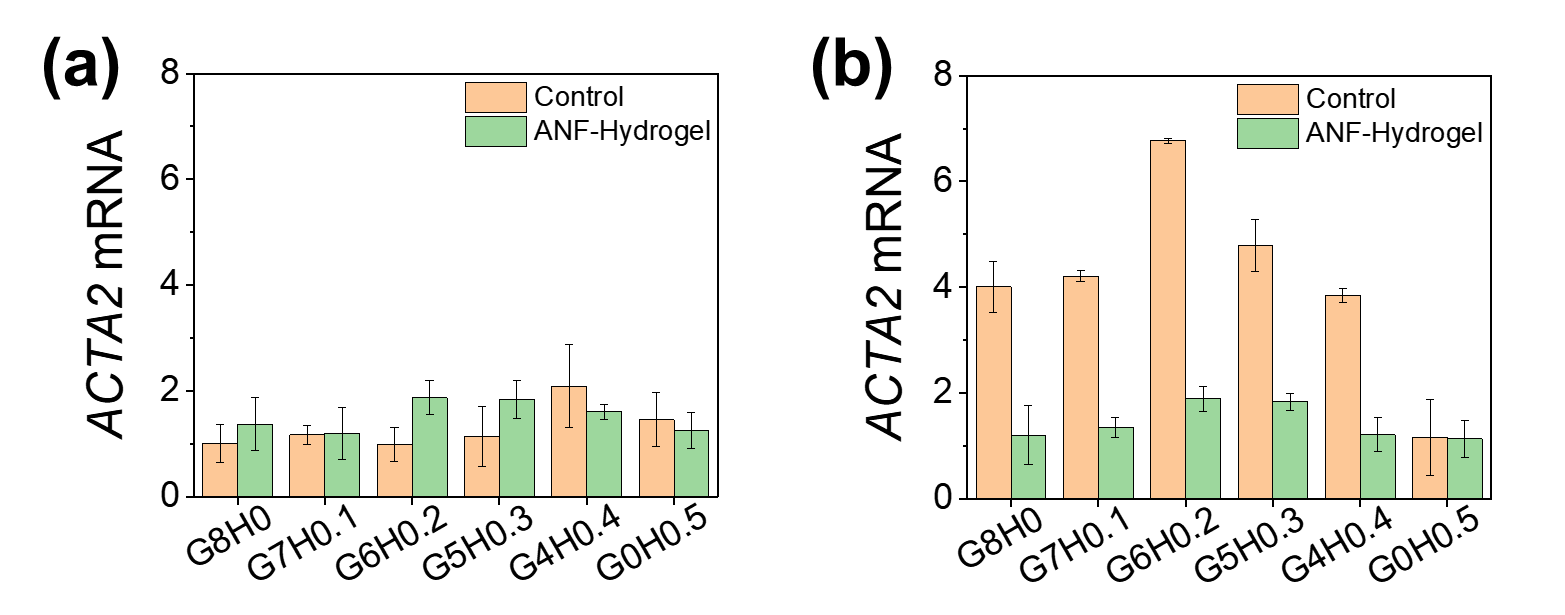


**Figure S5**. *ACTA2* mRNA expression levels from dermal fibroblasts in control hydrogel (without NF) and ANF-hydrogels with varying MHA concentrations hydrogels relative to those cultured on plastic culture plate, quantified by qRT-PCR: (a) without TGF-β, (b) with TGF-β.


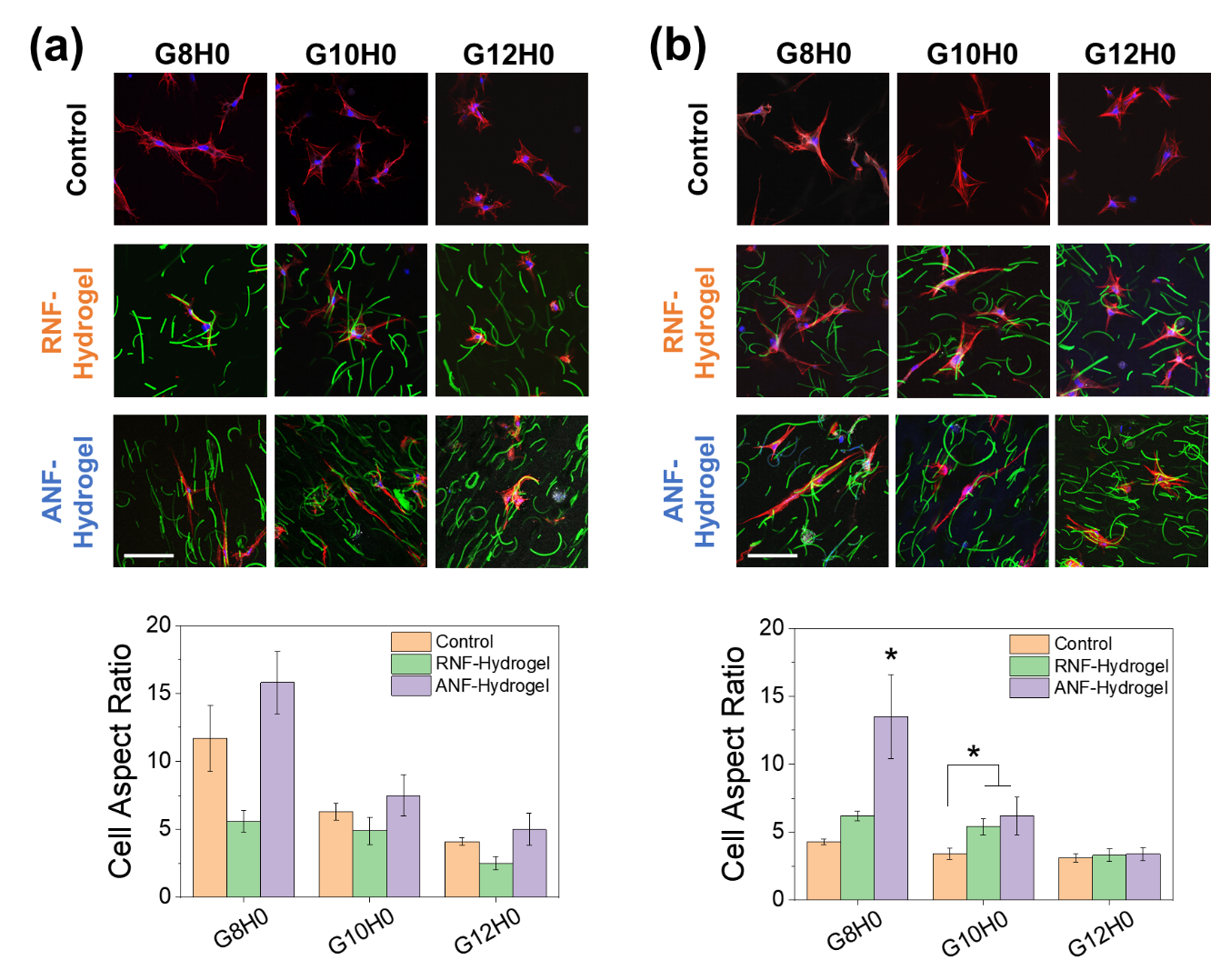


**Figure S6.** Representative fluorescent microscopic images of dermal fibroblasts in control hydrogels (without NF), RNF-hydrogels and ANF-hydrogels, and hydrogels without NF (scale bar: 10 µm); (a) normal culture, (b) supplemented with TGF-β. F-actin and nuclei of the cells and the nanofibers were fluorescently labeled. The cell aspect ratios were quantified by measuring the maximum and minimum lengths in (a) and (b) and presented in (c) and (d), respectively (**p*<0.05, when compared with other nanofiber conditions in the same hydrogel, *n*=10)


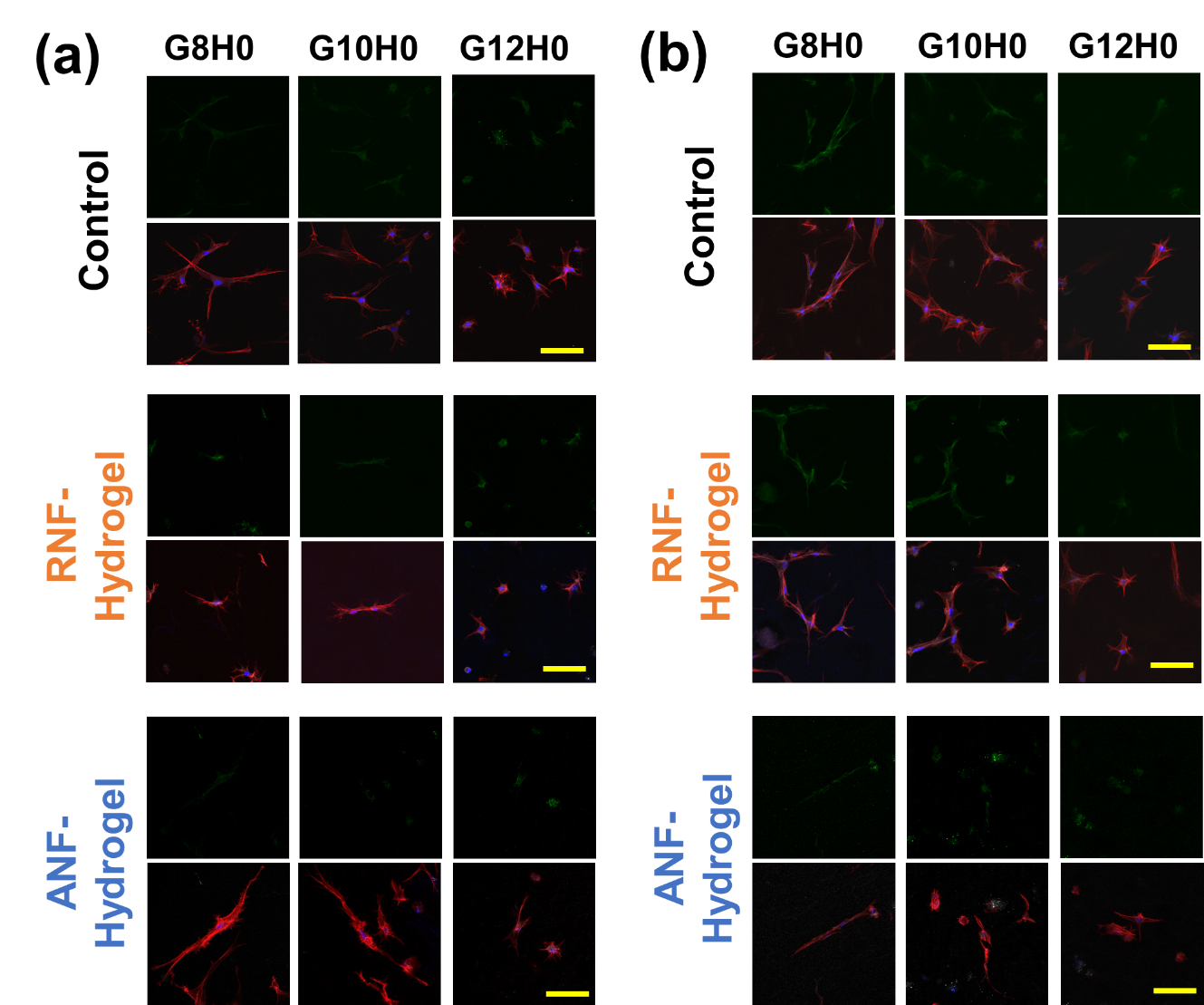


**Figure S7.** Representative fluorescent microscopic images of fibroblasts cultured in control hydrogel (without NF), RNF-hydrogels, and ANF-hydrogels with varying MGel concentrations (scale bar: 10 µm); (a) normal culture, (b) supplemented with TGF-β. Alpha smooth muscle actin (α-SMA) was fluorescently labeled (green). F-actin (red) and nuclei (blue) were also fluorescently labeled to visualize the overall cell morphology.
